# The incidence and nature of in-hospital adverse events: a systematic review

**DOI:** 10.1136/qshc.2007.023622

**Published:** 2008-06-02

**Authors:** E N de Vries, M A Ramrattan, S M Smorenburg, D J Gouma, M A Boermeester

**Affiliations:** 1 Department of Surgery, Academic Medical Centre, University of Amsterdam, The Netherlands; 2 Department of Pharmacy, Academic Medical Centre, University of Amsterdam, The Netherlands

## Abstract

**Introduction:**

Adverse events in hospitals constitute a serious problem with grave consequences. Many studies have been conducted to gain an insight into this problem, but a general overview of the data is lacking. We performed a systematic review of the literature on in-hospital adverse events.

**Methods:**

A formal search of Embase, Cochrane and Medline was performed. Studies were reviewed independently for methodology, inclusion and exclusion criteria and endpoints. Primary endpoints were incidence of in-hospital adverse events and percentage of preventability. Secondary endpoints were adverse event outcome and subdivision by provider of care, location and type of event.

**Results:**

Eight studies including a total of 74 485 patient records were selected. The median overall incidence of in-hospital adverse events was 9.2%, with a median percentage of preventability of 43.5%. More than half (56.3%) of patients experienced no or minor disability, whereas 7.4% of events were lethal. Operation- (39.6%) and medication-related (15.1%) events constituted the majority. We present a summary of evidence-based interventions aimed at these categories of events.

**Conclusions:**

Adverse events during hospital admission affect nearly one out of 10 patients. A substantial part of these events are preventable. Since a large proportion of the in-hospital events are operation- or drug-related, interventions aimed at preventing these events have the potential to make a substantial difference.

Adverse events (AEs) in hospitals are now widely agreed to be a serious problem, annually killing more people than breast cancer or AIDS.^1^ An AE is usually defined as an unintended injury or complication resulting in prolonged hospital stay, disability at the time of discharge or death and caused by healthcare management rather than by the patient’s underlying disease process.^2^ ^3^ Aside from the direct harm to the patient, AEs are a considerable financial burden to the healthcare system. In 1999, it was estimated that the total costs of preventable AEs in the USA lie between $17 billion and $29 billion annually.^4^

In recent years, the focus in thinking about AEs has shifted from the person approach—blaming individuals for errors—to the systems approach. The systems approach assumes that people will make mistakes, and that the system that surrounds them should provide a safety net for these mistakes. Therefore, efforts to eliminate AEs should be directed towards a particular system.^5^ This new approach has shifted the focus of the debate on AEs from the legal consequences associated with personal responsibility, to a more constructive point of view, clearing the way for thinking about solutions.

In the aftermath of the 2001 Institute of Medicine report “To err is human,”^1^ many large studies have been performed concerning AEs, some of them nationwide. Although many of these studies used similar methods, they report substantially different incidences. A general overview of data on in-hospital AEs is lacking.

To make the important step towards solutions, it is necessary to gain a more detailed understanding of this problem: what percentage of events is preventable, where do the majority of events happen and which type of event is the most frequent? This will enable identification of categories of AEs that are most susceptible to interventions to improve patient safety.

To gain an insight into the overall incidence, preventability, outcome and subdivision by location, provider and type of in-hospital AEs and the evidence related to relevant patient safety interventions, we conducted a systematic review of available data from the literature.

## METHODS

### Literature search

Two authors (ENV, MAB) independently performed a formal computer-assisted search of the medical databases Medline (January 1966 to February 2007), Cochrane and Embase (January 1980 to February 2007). Keywords used were “adverse events” and “preventable.” Clinical studies published in peer-reviewed journals in the English language were identified. A manual cross-reference search of the eligible papers was performed to identify additional relevant articles.

### Selection

In order to be able to reliably compare the data, we defined an AE as follows: an unintended injury or complication resulting in prolonged hospital stay, disability at the time of discharge or death and caused by healthcare management rather than by the patient’s underlying disease process. All studies that used this or a similar definition to evaluate the incidence of AEs in adult hospital patients and that included a minimum of 1000 patient records were eligible for inclusion. Studies that evaluated errors without linking them to outcomes and studies relying only on computerised screening data were excluded. Studies that evaluated specific types of AEs only (for example, adverse drug events only) and studies that evaluated specific populations (for example, ICU patients only) were excluded. No abstract publications without subsequent full-text published data were used. Disagreements about inclusion were resolved in a consensus meeting.

### Validity assessment

Two authors (ENV, MAB) independently assessed selected studies for methodology and endpoints. Information was extracted on the methods of data collection (prospective or retrospective), record selection and review, the time frame of included AEs and recorded interobserver variability. Primary endpoints were the incidence of AEs and the percentage of preventability. Secondary endpoints were adverse event outcome and subdivision by provider of care, location and type of event.

### Data collection

Data on incidence of AEs, preventability, outcome, location, provider of care and type of event were extracted. Whenever possible, raw data were used, and percentages were calculated. Extrapolations to state or country levels were not reproduced. Data on outcome, provider of care, location and type of event were grouped into common categories that the majority of articles used.

### Interventions

After analysis of the data yielded the categories of events that were responsible for the majority of adverse events, a computer-assisted search of Medline was performed to identify interventions relating to these categories of events. Only studies with a level of evidence of one or two were included.

### Statistical analysis

Medians and interquartile ranges (IQR) of incidence, preventability, and the different categories of outcome, location, provider of care and type of event were calculated using Statistical Package for the Social Sciences version 12.0 (SPSS, Chicago).

## RESULTS

### Article retrieval

**Figure 1:**
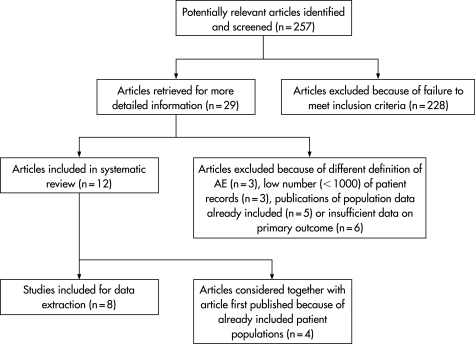
Flow chart of article retrieval. AE, adverse event.

The initial search yielded 257 articles (fig 1). After reviewing the titles and abstracts, 228 articles were excluded. These articles included reviews, studies on specific types of AEs only, for example adverse drug events, and studies in specific populations, for example children or ICU patients. Of the remaining 29 studies, another 17 were excluded after reviewing the full article. Three of these studies applied a different definition of an AE; one study used an observational approach and recorded only errors without linking them to outcomes;^6^ the other two studies used a computer-assisted approach to screen a large number of patient records for certain codes denoting complications.^7^ ^8^ Three studies were excluded because of an insufficient number of patient records; one of these studies used retrospective record review;^9^ the other studies would otherwise have been excluded due to methodological designs that differed from the large record review studies.^10^ ^11^ Five studies presented data of patient populations already included in other publications,^12^^–^16 and six studies presented insufficient data on the primary endpoint.^17^^–^22

### Included studies

We included 12 articles in the review. Of these articles, four reported additional data of patient populations that were already included.^23^^–^26 In this review, these articles were considered as one study together with the article first published, resulting in eight reviewed studies.

### Study characteristics

Characteristics of included studies are presented in tables 1 and 2. A total of 74 485 patient records were derived from the included studies. The number of hospitals per study ranged from 1 to 51 and the median number of patient records reviewed per study was 5162 (IQR 1542–14 569). All studies used a two-stage retrospective record review technique. Records were first screened by trained nurses; when positive for certain trigger criteria^27^ (for example an unplanned readmission, an adverse drug reaction or a hospital-acquired infection), the records were then reviewed by a physician to determine whether or not an AE had occurred. In two studies, the data from this approach were compared with voluntary reporting data.^28^ ^29^ Whether or not an event was caused by healthcare management (causality judgement) was measured on a six-point scale in six studies; a score of ⩾2^3^ ^30^ or ⩾4^2^ ^31^^–^33 was considered positive. The other two studies did not specify the details of the causality judgement.^28^ ^29^ Of the six studies that gave a judgement on preventability, four used a similar six-point scale; a score of ⩾4 was considered preventable.^3^ ^24^ ^28^ ^31^ The other two studies did not specify the details of the preventability judgement.^29^ ^33^ 

**Table 1:** Included studies

Reference	Year of publication	Country	No. of hospitals	No. of records	Inclusion period	Study design
1. Brennan *et al*^25^,^32^	1991	USA	51	30 121	1984	Retrospective record review
2. O’Neil *et al*^23^,^28^	1993	USA	1	3141	1990–1991	Retrospective record review and review of voluntary reporting data
3. Wilson *et al*^3^	1995	Australia	28	14 179	1992	Retrospective record review
4. Thomas *et al*^2^	2000	USA	28	14 700	1992	Retrospective record review
5. Vincent *et al*^26^,^33^	2001	UK	2	1014	1998	Retrospective record review
6. Davis *et al*^24^,^30^	2002	New Zealand	13	6579	1998	Retrospective record review
7. Baker *et al*^31^	2004	Canada	20	3745	2000	Retrospective record review
8. Sari *et al*^29^	2006	UK	1	1006	2004	Retrospective record review and review of voluntary reporting data

**Table 2:** Study characteristics

Reference	Population	Method of record selection	Method of review	More than one adverse event per patient?	Time frame of included events	Kappa value for interobserver agreement	Endpoints
1. Brennan *et al*^25^,^32^	Acute-care hospital patients (no psychiatric patients)	Random sample of hospitalisations from 51 hospitals	Two-stage record review: first, screening for one of 18 criteria by trained nurses; second, review by two physicians	Not specified	Occurred before and during and detected during index admission	0.61	Incidence, negligence, outcome, location, type of event
2. O’Neil *et al*^23^,^28^	Hospital patients	All admissions to the medical service of one hospital over a 4-month period	1. Two-stage record review: first, screening for one of 15 criteria by medical-record analysts; second, review by physician	No	Not specified	0.57	Incidence, preventability
			2. Reviewing reported incidents				
3. Wilson *et al*^3^	Acute-care hospital patients (no psychiatric or day-care patients)	Random sample of admissions from 28 hospitals	Two-stage record review: first, screening for one of 18 criteria by trained nurses; second, review by two medical officers	No	Occurred before* and during and detected during or after index admission	0.55	Incidence, preventability, outcome, provider of care, location, type of event
4. Thomas *et al*^2^	Hospital patients (no psychiatric, rehabilitation or drug/alcohol treatment patients)	Random sample of discharges from 28 hospitals	Two-stage record review: first, screening for one of 15 criteria by trained nurses; second, review by physician	No	Occurred before and during and detected during index admission	0.4	Incidence, negligence, outcome, provider of care, location, type of event
5. Vincent *et al*^26^,^33^	Acute hospital patients (general medicine, general surgery, orthopaedic surgery, obstetrics)	Records randomly drawn from two hospitals	Two-stage record review: first, screening for one of 18 criteria by trained nurses; second, review by clinician	Yes	Not specified	Not specified	Incidence, preventability, outcome, provider of care, type of event
6. Davis *et al*^24^,^30^	Hospital patients (no psychiatric, day-care or rehabilitation patients)	Random sample of admissions from 13 hospitals	Two-stage record review: first, screening for one of 18 criteria by trained nurses; second, review by medical officer	Not specified	Occurred before* and during and detected during index admission	0.47	Incidence, preventability, outcome, provider of care, location, type of event
7. Baker *et al*^31^	Hospital patients (no paediatric, psychiatric, obstetric or rehabilitative)	Random sample of admissions from 20 hospitals	Two-stage record review: first, screening for one of 18 criteria by trained nurses; second, review by physician	Yes	Occurred before and during and detected during or after index admission†	0.47/0.45/0.69‡	Incidence, preventability, outcome, provider of care, type of event
8. Sari *et al*^29^	Hospital patients (surgery, general medicine, elderly care, orthopaedics, urology, oncology, ENT, ophthalmology)	Random sample of admissions in one hospital	1. Two-stage record review: first, screening for one of 18 criteria by trained nurses; second, review by physician	Yes	Not specified	0.76	Incidence
			2. Reviewing reported incidents				

*Only included if (partly) responsible for index admission.

†Only included if occurred/was detected during a hospital admission.

‡Kappa values for interobserver agreement of judgement on injury/causation/preventability.

### Incidence, preventability and outcome

The data on incidence, preventability and outcome of case record review studies are shown in table 3. The median incidence of AEs was 9.2% (IQR 4.6–12.4%). The median percentage of AEs that was judged preventable was 43.5% (IQR 39.4–49.6%). Two studies judged negligence instead of preventability,^2^ ^32^ which was defined as AEs caused by a failure to meet standards reasonably expected of the average physician or institution. Negligence data were not considered in the calculation of the median percentage of preventability.

**Table 3 QHE-17-03-0216-t03:** Adverse events, preventability and outcome

Reference	Brennan *et al*^32^	O’Neil *et al*^23^,^28^	Wilson *et al*^3^	Thomas *et al*^2^	Vincent e*t al*^33^	Davis *et al*^24^,^30^	Baker *et al*^31^	Sari *et al*^29^	Median percentage (interquartile range)
No. of records	30 121	3141	14 179	14 700	1014	6579	3745	1006	–
No. of patients with at least one adverse event	1133 (3.8)	237* (7.5)	2353 (16.6)	475 (3.2)	110 (10.8)	850 (12.9)	255 (6.8)	110 (10.9)	9.2 (4.6 to 12.4)
No. of adverse events (if >1 adverse event per patient)	–	–	–	–	119 (11.7)	–	289 (7.7)	136 (13.5)	11.7 (7.7 to 13.5)
No. of preventable adverse events	–	103* (43.5)	1205 (51.2)	–	57 (47.9)	315 (37.1)	106 (41.6)	–	43.5 (39.4 to 49.6)
									
**Outcome**									
No or minor disability†	644 (56.8)	–	1073 (45.6)	253 (53.3)	73 (66.4)	524 (61.6)	161 (55.7)	–	56.3 (51.4 to 62.8)
Temporary disability‡	187 (16.5)	–	702 (29.8)	150 (31.6)	21 (19.1)	162 (19.0)	36 (12.5)	–	19.1 (15.5 to 30.3)
Permanent disability§	74 (6.5)	–	315 (13.4)	40 (8.4)	7 (6.4)	87 (10.2)	15 (5.2)	–	7.0 (6.1 to 11.0)
Death	154 (13.6)	–	112 (4.8)	31 (6.6)	9 (8.2)	38 (4.5)	46 (15.9)	–	7.4 (4.7 to 14.2)
Unknown	75 (6.6)	–	151 (6.4)	–	–	40 (4.7)	31 (10.7)	–	6.5 (5.1 to 9.7)

Numbers (percentages) except last column. Interquartile range = 25th to 75th percentile. Brennan: no. of records, number of AEs and percentages of outcomes were given. Percentage of AEs and numbers of outcomes were calculated. No number or percentage was given for preventability. No. of AEs with negligence (failure to meet standards reasonably to be expected) was 280 (24.7%). O’Neil: two different strategies were used. No. of records, number and percentage of AEs per strategy and percentage of preventability per strategy were given. Total number and percentage of AEs and total number and percentage of preventable AEs were calculated. Bates: no. of records, number of AEs and percentage of AEs were given. Wilson: no. of records, number and percentage of AEs, percentage of preventability and numbers were given. No. of preventable AEs and percentages of outcomes were calculated. Thomas: no. of records, number of AEs and percentages of outcomes were given. Percentage of AEs and numbers of outcomes were calculated. No number or percentage was given for preventability. Percentage of AEs with negligence (failure to meet standards reasonably to be expected) was 32.6 for Utah and 27.5 for Colorado. Vincent: no. of records, number and percentage of AEs, number and percentage of preventability and numbers and percentages of outcomes were given. Davis: no. of records, number and percentage of AEs, number and percentage of preventability and percentages of outcomes were given. Numbers of outcomes were calculated. Baker: no. of records, number of AEs, number and percentage of preventability and numbers and percentages of outcomes were given. Percentage of AEs was calculated. Sari: two different strategies were used, numbers and percentages in table are combined results. No. of records and number of AEs were given. Percentage of AEs was calculated.

*O’Neil *et al* used two different strategies. Bates *et al* evaluated the same population using the same methods for retrospective record review and reported another incidence. The numbers and percentages given here represent the mean of the combined results from the publication by O’Neil *et al* and the results by Bates *et al*. Idem for the preventability numbers.

†No disability or disability resolved within 1 month; contains Thomas’ categories of “emotional,” “insignificant” and “minor temporary” and Baker’s categories of “none” and “minimal.”

‡Contains Brennan’s categories of “disability resolved within 1–6 months” and “within 6–12 months,” Thomas’ category of “major temporary” and Baker’s categories of “impairment resolved within 1–6 months” and “within 6–12 months.”

§Contains Thomas’ categories of “minor permanent,” “significant permanent,” “major permanent” and “grave.”

Outcome data were divided into five categories: no or minor disability (resolved within 1 month), temporary disability (resolved within 1 year), permanent disability, death and unknown. The median percentage of AEs that led to no or minor disability was 56.3% (IQR 51.4–62.8%). Permanent disability was found in 7.0% (IQR 6.1–11.0%) of patients experiencing an AE, while 7.4% (IQR 4.7–14.2%) of AEs caused the death of the patient.[Table QHE-17-03-0216-t03]

### Providers of care

Providers of care were divided into three groups (table 4): surgical, containing all surgical professions, anaesthesiology, gynaecology and obstetrics; medicine, containing all internal specialties and paediatrics; and “other,” containing for example family practice, nursing and emergency medicine. The median proportion of AEs associated with surgical providers was 58.4% (IQR 54.5–70.9%) versus 24.1% (IQR 18.7–40.4%) for medical providers. 

**Table 4:** Adverse events classified by providers of care

Reference	Wilson *et al*^3^	Thomas *et al*^2^	Vincent *et al*^26^	Davis *et al*^24^	Baker *et al*^31^	Median percentage (interquartile range)
No. of adverse event/total no. of records	2353/14 179	475/14 700	119/1 014	850/6 579	289/3 745	–
Surgical	1375 (58.4)	298 (62.7)	94 (79.0)	489 (57.5)	149 (51.4)	58.4 (54.5 to 70.9)
Surgery not otherwise specified	–	219 (46.1)	–	–	–	–
General surgery	317 (13.5)	–	47 (39.5)	–	–	26.5 (13.5 to 39.5)
Orthopaedic surgery	285 (12.1)	–	40 (33.6)	–	–	22.9 (12.1 to 33.6)
Obstetrics	140 (5.9)	44 (9.2)	7 (5.9)	–	–	5.9 (5.9 to 9.2)
Gynaecology	134 (5.7)	32 (6.7)	–	–	–	6.2 (5.7 to 6.7)
Urology	86 (3.7)	–	–	–	–	–
Cardiac surgery	77 (3.3)	–	–	–	–	–
Vascular surgery	71 (3.0)	–	–	–	–	–
Otorhinolaryngology	59 (2.5)	–	–	–	–	–
Neurosurgery	57 (2.4)	–	–	–	–	–
Colorectal surgery	53 (2.3)	–	–	–	–	–
Plastic surgery	49 (2.1)	–	–	–	–	–
Anaesthesiology	47 (2.0)	3 (0.7)	–	–	–	1.4 (0.7 to 2.0)
Medicine	385 (16.4)	114 (24.1)	25 (21.0)	303 (35.7)	130 (45,0)	24.1 (18.7 to 40.4)
Internal medicine	150 (6.4)	110 (23.2)	–	–	–	14.8 (6.4 to 23.2)
Cardiology	118 (5.0)	–	–	–	–	–
Paediatrics	49 (2.1)	4 (0.9)	–	–	–	1.5 (0.9 to 2.1)
Gastroenterology	43 (1.8)	–	–	–	–	–
Medical oncology	25 (1.1)	–	–	–	–	–
Other	542 (23.0)	57 (11.9)	–	58 (6.8)	10 (3.6)	9.4 (4.4 to 20.2)
Family practice	147 (6.2)	21 (4.4)	–	–	–	5.3 (4.4 to 6.2)
Nursing	85 (3.6)	8 (1.7)	–	–	–	2.7 (1.7 to 3.6)
Emergency medicine	34 (1.4)	8 (1.7)	–	–	–	1.6 (1.4 to 1.7)
Ophthalmology	28 (1.2)	–	–	–	–	–
Radiology	–	5 (1)	–	–	–	–
Other	248 (10.5)	15 (3.1)	–	–	–	6.8 (3.1 to 10.5)
Unknown	51 (2.2)					

Numbers (percentages) except last column. Interquartile range = 25th to 75th percentile. Wilson: numbers and percentages were given. Thomas: percentages were given, and numbers were calculated. Vincent: numbers were given, and percentages were calculated. Davis: numbers and percentages were given. Baker: numbers were given in a crosstable with type of event. Because AEs could be attributed to more than one type of event, the total was 360. Numbers and percentages in this table were calculated as a percentage of the total number of AEs.

### Locations

Table 5 shows the various locations where AEs took place. For all AEs, 80.8% (IQR 75.6–83.2%) were encountered in hospital, versus 14.9% (IQR 12.9–18.7%) out of hospital before admission or after discharge. The majority of events were seen in the operating room (41.0% (IQR 39.5–45.8%)) or the patient’s room (24.5% (IQR 21.6–26.5%)). By contrast, only 3.1% (IQR 2.7–3.5%) of AEs were located in the complex environment of the intensive care unit. The emergency room accounted for 3.0% (IQR 2.9–3.0%) of AEs.

**Table 5:** Adverse events classified by location

Reference	Brennan *et al*^25^	Wilson *et al*^3^	Thomas *et al*^2^	Davis *et al*^24^	Median percentage (interquartile range)
No. of adverse events/total no. of records	1133/30 121	2353/14 179	475/14 700	850/6579	–
In hospital	920 (81.2)	1741 (74.0)	398 (83.8)	683 (80.4)	80.8 (75.6 to 83.2)
Operating room	465 (41.0)	1077 (45.8)	188 (39.5)	–	41.0 (39.5 to 45.8)
Patient’s room	300 (26.5)	577 (24.5)	103 (21.6)	–	24.5 (21.6 to 26.5)
Emergency room	33 (2.9)	–	14 (3.0)	–	3.0 (2.9 to 3.0)
Labour and delivery room	32 (2.8)	87 (3.7)	31 (6.5)	–	3.7 (2.8 to 6.5)
Intensive care unit	31 (2.7)	–	17 (3.5)	–	3.1 (2.7 to 3.5)
Radiology	23 (2.0)	–	–	–	–
Cardiac catheterisation laboratory	10 (0.9)	–	20 (4.2)	–	2.6 (0.9 to 4.2)
Ambulatory care unit	9 (0.8)	–	–	–	–
Procedure room	–	–	16 (3.4)	–	–
Other	19 (1.7)	–	10 (2.2)	–	2.0 (1.7 to 2.2)
Out of hospital	156 (13.8)	297 (12.6)	76 (16.0)	167 (19.6)	14.9 (12.9 to 18.7)
Physician’s office	87 (7.7)	200 (8.5)	33 (7.0)	54 (6.4)	7.4 (6.6 to 8.3)
Patient’s home	31 (2.7)	56 (2.4)	16 (3.4)	45 (5.3)	3.1 (2.5 to 4.8)
Ambulatory care unit	16 (1.4)	–	–	11 (1.3)	1.4 (1.3 to 1.4)
Nursing home	10 (0.9)	41 (1.7)	3 (0.6)	32 (3.8)	1.3 (0.7 to 3.3)
Day surgery	–	–	6 (1.2)	–	–
Private hospital	–	–	–	17 (2.0)	–
Other	12 (1.1)	–	18 (3.8)	8 (0.9)	1.1 (0.9 to 3.8)
Unknown	58 (5.1)	315 (13.4)	1 (0.3)	–	5.1 (0.3 to 13.4)

Numbers (percentages) except last column. Interquartile range = 25th to 75th percentile. Brennan: percentages were given, and numbers were calculated. Wilson: numbers and percentages were given. Thomas: percentages were given, and numbers were calculated. Davis: percentages were given, and numbers were calculated.

### Type of event

Finally, the AEs were classified according to type of event (table 6). In three studies an AE could be attributed to more than one category,^3^ ^24^ ^31^ whereas in the other studies, the types of events were mutually exclusive. Importantly, approximately 50% of AEs are operation- or drug-related: 39.6% (IQR 31.5–50.2%) and 15.1% (11.9–20.4%), respectively. By contrast, anaesthesia-related events formed only 2.0% (IQR 1.2–3.7%) of AEs.

**Table 6:** Adverse events classified by type of event

Reference	Brennan *et al*^25^	Wilson *et al*^3^	Thomas *et al*^2^	Vincent *et al*^26^	Davis *et al*^24^	Baker *et al*^31^	Median percentage (interquartile range)
No. of adverse events/total no. of records	1133/30 121	2952/14 179	475/14 700	118/1 014	1060/6 579	360/3 745	–
Operation-related	599 (52.9)	1159 (49.3)	213 (44.9)	40 (33.9)	258 (24.3)	123 (34.2)	39.6 (31.5 to 50.2)
Drug-related	178 (15.7)	249 (10.6)	92 (19.3)	17 (14.4)	130 (12.3)	85 (23.6)	15.1 (11.9 to 20.4)
Diagnostic	79 (7.0)	314 (13.3)	33 (6.9)	5 (4.2)	85 (8.0)	38 (10.6)	7.5 (6.2 to 11.3)
Therapeutic	62 (5.5)	276 (11.7)	21 (4.4)	–	89 (8.4)	–	7.0 (4.7 to 10.9)
Procedure*	88 (7.8)	197 (8.4)	64 (13.5)	6 (5.1)	82 (7.7)	26 (7.2)	7.8 (6.7 to 9.7)
Fall/fracture	38 (3.4)	192 (8.2)	8 (1.7)	–	–	8 (2.2)	2.8 (1.8 to 7.0)
Postpartum/obstetric	18 (1.6)	126 (5.4)	17 (3.6)	–	–	1 (0.3)	2.6 (0.6 to 5.0)
Anaesthesia-related	13 (1.1)	51 (2.2)	6 (1.3)	6 (5.1)	–	7 (2.0)	2.0 (1.2 to 3.7)
Neonatal	29 (3.0)	30 (1.3)	15 (3.1)	–	–	–	3.0 (1.3 to 3.1)
System†/other	29 (3.0)	358 (15.2)	7 (1.5)	–	416 (39.3)	29 (8.1)	8.1 (2.3 to 27.3)
Ward management	–	–	–	30 (25.4)	–	–	–
Discharge	–	–	–	14 (11.9)	–	–	–
Other clinical management	–	–	–	–	–	43 (11.9)	–

Brennan: numbers were given, percentages were calculated. Wilson: numbers and percentages were given. Thomas: percentages were given, and numbers were calculated. Vincent: numbers were given, and percentages were calculated. Davis: numbers and percentages were given. Baker: numbers were given, and percentages were calculated. Numbers (percentages) except last column. Interquartile range = 25th to 75th percentile.

*Medical procedure such as coronary angiography or endoscopy.

†Contains: defective equipment or supplies, inadequate reporting or communication, inadequate staffing, training or supervision, no protocol/failure to implement protocol.

### Patient safety interventions

Table 7 gives an overview of the top level (level of evidence 1 and 2) of evidence-based interventions directed towards the major types of adverse events: operation- and medication-related events. The operation-related interventions include a number of medical interventions such as perioperative beta-blockade and antibiotic prophylaxis. In addition, interventions such as training programmes for laparoscopy and a medical emergency team are mentioned. The medication-related practices include bar code technology and computerised physician order entry systems.

**Table 7:** Interventions related to operation- and drug-related events

Type of event	Intervention		Highest-level study example	Level of evidence
Operation-related	Localising care to high-volume centres		Halm *et al*. Is volume related to outcome in health care? A systematic review and methodologic critique of the literature. *Ann Intern Med* 2002^34^	2A
	Training programmes for laparoscopic procedures		Scheeres *et al*. Animate advanced laparoscopic courses improve resident operative performance. *Am J Surg* 2004^35^	2B
	Medical emergency team		Bellomo *et al*. Prospective controlled trial of effect of medical emergency team on postoperative morbidity and mortality rates. *Crit Care Med* 2004^36^	2B
	Ultrasound guidance of central vein catheterisation		Randolph *et al*. Ultrasound guidance for placement of central venous catheters: a meta-analysis of the literature. *Crit Care Med* 1996^37^	1A
	Prevention of surgical site infections:	Antibiotic prophylaxis	Song *et al*. Antimicrobial prophylaxis in colorectal surgery: a systematic review of randomized controlled trials. *Br J Surg* 1998^38^	1A
		Peri-operative normothermia	Kurz *et al*. Perioperative normothermia to reduce the incidence of surgical-wound infection and shorten hospitalization. *N Engl JMed* 1996^39^	1B
		Supplemental oxygen	Greif *et al*. Supplemental perioperative oxygen to reduce the incidence of surgical-wound infection. *N Engl J Med* 2000^40^	1B
		Glucose control in diabetics	Furnary *et al*. Clinical effects of hyperglycemia in the cardiac surgery population: the portland diabetic project. *Endocr Pract* 2006^41^	2B
	Perioperative beta blockers		Devereaux *et al*. How strong is the evidence for the use of perioperative beta blockers in non-cardiac surgery? Systematic review and meta-analysis of randomised controlled trials. *BMJ* 2005^42^	1A
Drug-related	Computerised Physician Order Entry (CPOE) and Clinical Decision Support System (CDSS)		Overhage *et al*. A randomized trial of “corollary orders” to prevent errors of omission. *J Am Med Inform Assoc* 1997^43^	1B
	Clinical pharmacist consultation services		Leape *et al*. Pharmacist participation on physician rounds and adverse drug events in the intensive care unit. *JAMA* 1999^44^	2B
	Bar code technology in pharmacy		Poon *et al*. Medication dispensing errors and potential adverse drug events before and after implementing bar code technology in the pharmacy. *Ann Intern Med* 2006^45^	2B
	Patient self-management of anticoagulation		Cromheecke *et al*. Oral anticoagulation self-management and management by a specialist anticoagulation clinic: a randomized crossover comparison. *Lancet* 2000^46^	1B

## DISCUSSION

We conducted a systematic review to gain an insight into the overall incidence, preventability and outcome of adverse events and added information about location, provider and type of events. Despite the enormous amount of recent attention for patient safety, such systematic compiling of all available evidence on the subject is lacking to date.

This systematic review included eight studies from the USA, Canada, the UK, Australia and New Zealand. The median overall incidence of adverse events was 9.2%, and almost half of these events were regarded as preventable. The majority of events were associated with a surgical care provider, and more than half of events were operation- or drug-related.

Although all included studies used the same definition, incidences of adverse events varied considerably. In 2000, a comparison was made between the Utah/Colorado study (incidence 3.2%) and the Australian study (incidence 16.6%), and a number of possible reasons for this difference were provided.^47^ ^48^ There were a number of methodological differences between the studies, such as a lower threshold for defining causation in the Australian study and inclusion of some types of events in one study that were excluded in the other. Aside from these differences, the authors suggest that the disparity might be due to differences in quantity and methods of documentation between Australia and the USA, and different perspectives of the two studies (medicolegal versus quality-improvement). These considerations may also apply to the other studies included in this review. For example, both studies that were performed from a medicolegal point of view^2^ ^32^ reported considerably lower incidences than the other studies, performed from a quality-improvement point of view.^3^ ^28^–^31 33^ Furthermore, not all studies employed the same time frame for included events. Out-of-hospital events were included in only a few studies. Some studies only recorded one adverse event per patient record, whereas others did not enforce this restriction. These methodological differences may well, at least in part, account for the difference between the recorded incidences.

Retrospective record review has been criticised for a number of reasons. As it relies heavily on patient records, it is dependent on the quality of documentation. If adverse events are not documented properly, they will not be detected by this method. Furthermore, only those adverse events are detected that result in one of the trigger criteria of the review method. Finally, in retrospective record review, the interobserver variability is very high, especially with regard to the judgements on causality and preventability.^49^ The studies included in this review show moderate interobserver agreement scores, illustrating this drawback of retrospective record review.

Aside from the fact that the conclusions from this review are based solely on retrospective record review studies, and as such, most likely represent an underestimation of the problem, there are a number of other limitations of this systematic review, the most important one being the heterogeneity of the included studies. Although most studies used roughly the same methods, the details differed considerably. For example, the studies from Australia and New Zealand applied a lower threshold for causation than the other studies. The time frame of included events also differed between studies: the studies from the United States did not include events discovered after discharge, whereas the other included studies did. Because differences in methodology and perspective may lead to different numbers of recorded AEs,^47^ ^48^ we must proceed with caution when drawing conclusions from the combined data from these studies. Apart from differences in methodology between the included studies, our strict inclusion criteria potentially may have caused us to exclude interesting studies. The three studies we excluded because of an insufficient number of patient records evaluated, when combined, 1607 records, amounting to 2% of all records included in this systematic review (more than 74 000 records). The two excluded studies that used a computer-assisted approach^7^ ^8^ reported incidences of adverse events that were slightly lower (8.3% and 6.9%) than the median incidence of the studies included in the present review. This approach is much less time-consuming than the retrospective record review, but a drawback is that it cannot make judgements on causation or preventability.

Much attention is being devoted to finding solutions to improve patient safety. In 2005, the authors of some of the largest adverse event studies advocated the implementation of selected evidence-based practices that have a potential for large impact.^50^ When looking at the classification of events as demonstrated in this review, operation-related and drug-related events together comprise the majority. It would thus be logical to concentrate funds and efforts on evidence-based interventions aimed at reducing these events. In addition to the evidence-based interventions reviewed here, there are a number of other interventions that seem promising but warrant further research to prove their value. This includes interventions derived from the aviation industry, such as crew resource management and the use of checklists in the operating room.

In conclusion, adverse events during hospital admission are a serious problem, occurring in approximately 9% of all admitted patients and leading to a lethal outcome in 7% of cases. Since a large portion of the adverse events are operation- or drug-related, and almost half of these events are preventable, funds and efforts should be concentrated on interventions aimed at reducing these types of events.
